# Disparities in Service and Clinical Outcomes in State-Wide Advanced Practice Physiotherapist-Led Services

**DOI:** 10.3390/healthcare9030278

**Published:** 2021-03-03

**Authors:** Maree Raymer, Louise Mitchell, Peter Window, Michelle Cottrell, Tracy Comans, Shaun O’Leary

**Affiliations:** 1 Department of Physiotherapy, Metro North Hospital Health Service, Royal Brisbane and Women’s Hospital, Brisbane, QLD 4029, Australia; maree.raymer@health.qld.gov.au (M.R.); Peter.Window@health.qld.gov.au (P.W.); Michelle.Cottrell@health.qld.gov.au (M.C.); s.oleary@uq.edu.au (S.O.); 2 Department of Health Queensland, Epidemiology and Research, Aboriginal and Torres Strait Islander Health Division, Brisbane, QLD 4001, Australia; Louise.Mitchell@health.qld.gov.au; 3 Centre for Health Services Research, Faculty of Medicine, The University of Queensland, Brisbane, QLD 4106, Australia; 4 School of Health and Rehabilitation Sciences, The University of Queensland, Brisbane, QLD 4072, Australia

**Keywords:** advanced practice, outcomes, outpatients, service variation, neurosurgery, orthopaedics, physiotherapy

## Abstract

This study explored variations in the primary service and clinical outcomes of a state-wide advanced practice physiotherapist-led service embedded in public medical specialist orthopaedic and neurosurgical outpatient services across Queensland, Australia. An audit of the service database over a six-year period was taken from 18 service facilities. The primary service and clinical outcomes were described. Variations in these outcomes between facilities were explored with a regression analysis adjusting for known patient- and service-related characteristics. The findings showed substantial positive impacts of the advanced practice model across all facilities, with 69.4% of patients discharged without a need for medical specialist review (primary service outcome), consistent with 68.9% of patients reporting clinically important improvements in their condition (primary clinical outcome). However, 15 facilities significantly varied from the state average for the primary service outcome (despite only three facilities varying in the primary clinical outcome). While this disparity in the primary service outcomes appears to be influenced by potentially modifiable differences in the service-related processes between facilities, these process differences only explained part of the variation. This study described the subsequent development of a new, more comprehensive set of service evaluation metrics to better inform future service planning.

## 1. Introduction

The Neurosurgical and Orthopaedic Physiotherapy Screening Clinic and Multi-disciplinary Service (N/OPSC & MDS) is an advanced practice physiotherapist-led model of care embedded in public hospital specialist orthopaedic and neurosurgical outpatient services across Queensland, Australia [1]. Patient referrals to these specialist medical outpatient services are initially triaged by either an N/OPSC & MDS advanced musculoskeletal physiotherapist (referred to herein as the “service leader”) or medical specialist (varies across facilities). Referrals considered appropriate to be directed to the physiotherapist-led service are usually patients with nonurgent musculoskeletal conditions (including neurosurgical patients with musculoskeletal conditions, e.g., neck and back disorders) potentially amenable to nonsurgical management. These eligible patients are referred to the N/OPSC & MDS for an initial assessment with the service leader. Depending on the initial assessment findings, a review by a medical specialist may be recommended (expedited, in some cases) for some patients (e.g., potentially serious conditions or surgical consideration required). Other patients may be discharged immediately for a variety of reasons (e.g., no longer seeking care or resolved or self-managed conditions), while the majority of patients will be referred for a trial of nonsurgical management [2]. All N/OPSC & MDS across the state follow a common model of care. Patient management within the service is individualised and may be multidisciplinary (e.g., physiotherapy, psychology, dietetics, occupational therapy, and pharmacy, as required) to pragmatically address the mix of biopsychosocial factors potentially underlying each individual patient’s chronic musculoskeletal condition [3,4]. The service leader reviews the progress as required, collaboratively deciding with the patient if further treatment is justified, if progress is adequate to warrant discharge from the service, or if medical specialist consultation is still needed.

The service assists patients in achieving meaningful clinical improvement in their condition (primary clinical outcome), minimising the proportion of patients requiring medical specialist review (primary service outcome). This primary service outcome has subsequent benefits for overburdened medical specialist orthopaedic and neurosurgical outpatient services. The initial success of this primary service outcome [1] underpinned the progressive rollout of the N/OPSC & MDS across Queensland, Australia (18 public hospitals and one community-based facility). This reflects that the majority of patients referred to specialist medical outpatients do not require surgery [5] and that less than five percent of patients discharged from the service present again for the same condition to specialist medical services within 12 months [6]. However, preliminary audits suggest variations in outcomes exist between service facilities, despite all facilities following a common model of care. N/OPSC & MDS facilities vary in size, personnel, patient demographics, and clinical populations (e.g., spine and upper and lower limbs) [7], as well as internal processes regarding triage and interprofessional communication. While variations to suit local health service settings and priorities is inevitable [8], the identification of potentially modifiable sources of variations in the outcomes between N/OPSC & MDS facilities is critical to maximising service impacts, providing information for future service planning [8] and the provision of equitable and efficient healthcare [9].

The overarching aim of this study was to investigate variation in outcomes between N/OPSC & MDS facilities to better inform future refinement of the state-wide service. Three specific objectives were set. The first objective was to evaluate the state-wide service and clinical outcomes of the N/OPSC & MDS over a six-year period. The second objective was to identify the extent and nature of the variation in these outcomes between facilities using the recommended methods [8]. Specifically, the nature of the variation was explored by determining if the currently collated N/OPSC & MDS evaluation metrics (service- and patient-related variables) could explain any observed variation in the outcomes between facilities. In particular, we wished to determine if any observed variation simply reflected differences in patient-related characteristics (demographic, social, global health, and clinical condition) between facilities or could instead be explained by differences in the service-related processes that could potentially be addressed in future service planning. The third objective was to interpret the findings from the perspective of future service evaluation and the potential need for a refined set of N/OPSC & MDS metrics.

## 2. Materials and Methods

### 2.1. Design

An audit of the N/OPSC & MDS Measurement Analysis and Reporting System database for the period 1 July 2012 to 10 April 2017 was undertaken from 18 eligible service facilities. This project received ethical approval by the institute’s ethical review committee (HREC/17/QRBW/154).

### 2.2. Audit and Data Extraction

#### 2.2.1. Primary Outcomes

The primary service outcome was a discharge pathway, dichotomised as either discharged from the service with no specialist medical review required (Discharged) or reinstated for specialist medical review (Specialist RV). The primary clinical outcome was dichotomised as achieving (Responder) or not achieving (Non-Responder) a clinically meaningful change in the presenting condition based on an 11-point Global Rating of Change (GROC) scale, with scores between +2 to +5 reflecting a Responder and scores between −5 to +1 reflecting a Non-Responder [10].

#### 2.2.2. Secondary Outcomes and Explanatory Variables

The secondary service- and patient-related outcomes and potential variation explanatory variables are listed in Table 1.

### 2.3. Data Analysis

Data was cleaned, coded, and quality-checked. All analyses were undertaken using SPSS v22 (IBM Corp, Armonk, NY, USA). Descriptive statistics were calculated to report the primary service and clinical outcomes, as well as the secondary service- and patient-related outcomes and explanatory variables (Study Aim 1). Paired *t*-tests and published Minimally Clinically Important Differences (MCID) (Table 1) where available were used to further explore secondary patient-related clinical outcomes.

Hierarchical binomial logistic regression analyses were then conducted to explore variation of service and clinical outcomes between facilities (Study Aim 2). Primary service (Discharged and Specialist RV) and clinical (Responder and Non-Responder) outcomes (dependent variables) were modelled separately to assess their relationships with facilities and the potential explanatory service-related variables (independent variables), while additionally accounting for influences of the patient-related explanatory variables (Aim 2). Potential service- and patient-related variable multicollinearity issues were firstly evaluated using Pearson’s (continuous normally distributed data) and Spearman’s rho (non-normally or categorical data) coefficients [27] to determine if moderate (r_s_ = 0.4–0.6) or strong (r_s_ = 0.7–0.9) correlations [28,29] were evident between variables. Where there was risk of multicollinearity, only one variable was selected (investigator’s choice based on clinical and service reasoning) to be carried forward to the final model.

In regression Model 1, the uncorrected relationship between the outcome and facility was evaluated. For both outcomes (service and clinical), the facility closest to the state average was coded as the “Referent” in SPSS. In Model 2, following the SPSS recommendations for hierarchical regression analyses [30], patient-related variables were entered, observing firstly their impact on the relationship between outcome and facility and, secondly, ensuring their potential influence on the impact of the explanatory service-related variables of interest (entered in the subsequent Model 3) were accounted for. As it was intended to determine the overall impact of the service-related variables, they were all entered together. Alpha was set at 0.05 for all statistical analyses.

## 3. Results

There were 29,319 eligible client records retrieved spanning six years (2012–2017). There were high rates of completion (>90%) of the service-related variables, as well as the patient sociodemographic measures, with minimal variations in data completeness across facilities. There was a lower completion rate for the primary (GROC, 55% completion) and secondary (completion rate range 33–80% at baseline and 24–64% at discharge) clinical outcome measures. Approximately 39% of patients were either discharged at their initial N/OPSC & MDS assessment or did not require review by the service leader and, therefore, were not eligible to complete the discharge clinical outcomes.

### 3.1. State-Wide N/OPSC & MDS Outcomes (Aim 1)

Across all the facilities, 69.4% of discharged patients did not require a medical specialist review (Primary service outcome), although this outcome varied across conditions, as shown in Table 2. As shown in Table 3, patients with spinal conditions represented the greatest proportion of patients managed (43.9% of cases), followed by patients with lower limb (32.7%) and upper limb (23.4%) conditions. Across all facilities, 68.9% of patients reported a clinically meaningful response to management within the N/OPSC & MDS, varying from 65.7% to 74.1% across conditions.

### 3.2. Variation in Outcome Measures between Facilities (Aim 2)

The hierarchical binomial regression modelling findings for the primary service (Discharged) and clinical (Responder) outcomes are shown in Table 4 and Table 5. The preliminary analyses showed many of the patient-related variables (Pain Severity, PSEQ, ODI, NDI, QDASH, and LEFS) to be significantly correlated (Spearman’s rho 0.35–0.69, *p* < 0.001). To avoid multicollinearity in the multivariable model, only the Pain Severity and QOL variables were selected to be carried through to the multivariate analysis based on their relevance to all the conditions (investigator’s judgement). The Outpatient Service and Condition Managed variables were also significantly related (Spearman’s rho 0.51, *p* < 0.001), as were the Management Duration and Review Appointments variables (Spearman’s rho 0.58, *p* < 0.001). Therefore, only the Condition Managed and Management Duration variables, respectively, were included in the multivariate analysis. The Box-Tidwell procedure [31] was performed using the variables remaining in the final models and the logit of the dependent variables (Pathway outcome and Clinical outcome). Both Age (*p* < 0.001) and Waiting Time (*p* < 0.001) demonstrated nonlinearity with the logit of the Clinical outcome (Responder/Non-responder) and were subsequently recoded to categorical variables for both regression models.

#### 3.2.1. Discharge Pathway (Primary Service Outcome)

The three progressive hierarchical binomial regression models for the primary service outcome of Discharge Pathway (reference: returned to specialist outpatients waitlist) are shown in Table 4 (Clinic 1 was coded as the Referent). In Model 1, 14 facilities are seen to be significantly different to the Referent, reducing to 10 facilities in Model 2 (adjusted for patient-related variables) and increasing to 15 facilities in Model 3 (adjusted for service-related variables). The significant service variables in the final model included Waiting Time, Management Duration, Triage Category, Non-attendance, and Medical Specialist Input during N/OPSC management.

No outliers were evident for the primary service outcome based on the studentised residual range (SD) (−2.73 to 2.50 (0.98)) being within accepted parameters (≤−3.61, ≥3.61) based on 13 predictor variables in the final models [32]. The logistic regression model was statistically significant, χ^2^(43) = 3681, *p* < 0.001. The model explained 32% (Nagelkerke R^2^) of the variance in the pathway outcome and correctly classified 76.8% of cases. One facility (Facility 16) had insufficient numbers and was excluded from the analyses for Models 2 and 3.

#### 3.2.2. GROC (Primary Clinical Outcome)

The three progressive models of the hierarchical binomial regression for the primary clinical outcome of GROC (reference: nonresponse to management) are shown in Table 5 (Clinic 10 was coded as the Referent). In Model 1, 13 facilities were significantly different from the Referent, reducing to four facilities in Model 2 (adjusted for the patient-related variables) and reducing to three facilities in Model 3 (adjusting for service-related variables). The significant service variables in the final model included Management Duration, Triage Category, Non-attendance, and Medical Specialist Input.

No outliers were evident for the primary clinical outcome, according to the studentised residual (range (SD) −2.63 to 2.02 (1.04)) [32]. The logistic regression model was statistically significant, χ^2^(42) = 879, *p* < 0.001. The model explained 18.1% (Nagelkerke R^2^) of the variance in the pathway outcomes and correctly classified 73.6% of cases. One facility (Facility 16) had insufficient numbers and was excluded from the analyses for Models 2 and 3.

## 4. Discussion

Over the 2012–2017 audit period, nearly 70% of patients discharged from management within the state-wide advanced physiotherapist-led N/OPSC & MDS did not require a specialist medical consultation (Primary Service Outcome). This is substantial from a specialist waitlist resource management perspective, given also that less than five percent of patients discharged by the service present again to specialist medical services within 12 months [6]. Furthermore, 69% of patients for whom clinical outcomes were received reported a clinically meaningful improvement in their condition (GROC, Primary Clinical Outcome). In context, these are notable primary service and clinical outcomes given the generally high levels of pain severity (average pain score 58/100) and disability (condition-specific index averaging 41–51/100) and low levels of function (PSFS 4/10) reported by patients at the initial consultation. In summary, these state-wide findings are consistent with earlier studies demonstrating the substantial impact the N/OPSC & MDS model of care delivers in managing orthopaedic and neurosurgical demands in Queensland’s public hospitals [7,33]. The future challenge will be in implementing the optimal scale and mix of specialist medical and advanced physiotherapist-led services to address demands at the various public hospital facilities [1,33,34].

The most critical finding of the study, though, indicated the full impact of the N/OPSC & MDS model in managing orthopaedic and neurosurgical demands in these public hospitals may not as yet be realised. Fifteen facilities were observed to be significantly different to the referent facility in their primary service outcome of a discharge pathway (Table 3). While adjustments for patient-related characteristics initially reduced the variations (14 to 10 facilities in Model 2), adjustments for the service-related variables inflated the variations between facilities (from 10 to 15 facilities in Model 3). The significant service-related variables in this final model included the duration of the management period, the initial triage category, patient non-attendance to review appointments, and medical specialist input during the management period. Potentially, changes in service planning may address these significant service-related variables, although some may be challenging to modify, given that they may reflect differences in organisational procedures at different health services. While some patient-related variables also remained significant in the final model (Age, Gender, Condition Managed, QOL, and Pain Severity at the initial consultation), from a service planning perspective, these are not modifiable factors. The most notable observation, though, was the remaining level of uncertainty regarding other potential sources of facility variation (model estimated to just explain approximately 32% of the variance) in the discharge outcomes. This relatively low level of explained variance strongly suggests that the currently collated N/OPSC & MDS service- and patient-related evaluation metrics are not sufficient to comprehensively explain the observed variations between facilities. Instead, future service evaluations will need to capture a broader suite of patient case mix and service evaluation metrics to better explain the variation in the discharge pathway.

In contrast, the primary clinical outcome only varied significantly at three facilities compared to the referent facility when the model was adjusted for the known patient- and service-related variables. Similar to the findings exploring the service outcome variation, the significant but potentially modifiable service-related variables in the final model included waiting time, management period, the initial triage category, patient non-attendance to review appointments, and medical specialist input during the management period. Collectively, the findings suggest that addressing the variation in these significant service-related variables may reduce the facility variation for both the primary service and clinical outcomes. Similar to the primary service outcome model, the estimated strength of this model was modest (<20% explained variance), further indicating a broader suite of measures needs to be investigated in future service evaluations.

While some variation may be inevitable between facilities, variation potentially reflects suboptimal service provisions [35]. This, in turn, may result in inequitable clinical outcomes and the inefficient use of healthcare resources, highlighting the opportunity for further quality improvement [8]. Overall, these findings have provided information for future service evaluations (Study Aim 3), resulting in a revised set of standardised state-wide metrics. These include additional patient/demographic variables, service-related variables, and changes in the Patient-Reported Outcome Measures (PROMs) collected. Details of the new standardised dataset and performance indicators can be found in Appendix A. In summary, the collective findings of this current study, reviews of relevant epidemiological [36,37,38], PROMs [39,40,41], and chronic disease database (national and international) literature [42,43,44], together with information derived from consultations and collaborations with the N/OPSC & MDS facilities, underpinned the inclusion of the revised metrics. In particular, facilities were asked to consider their local operational processes concerning factors such as triage, medical specialist case discussions, and patient non-attendance, as these variables were observed in this study to have the strongest influence (odds ratios (OR) as shown in Table 3) on patients progressing to medical specialist consultations. This collaborative work also resulted in the addition of other service-related variables (e.g., reason for medical consultant input and multidisciplinary referral patterns, including funding sources, referrals for investigations and interventions, and the use of telehealth during an episode of care). A follow-up study is planned to examine if this new dataset can better explain facility variation in outcomes compared to those observed in this current study.

Despite such a large sample size, there were still some limitations of this study, particularly the amount of missing data for clinical outcome measures and some variations in the outcomes recorded at different facilities. The findings for the primary clinical outcome regression model may also be limited due to the use of the GROC measures to dichotomise the outcomes. However, the service uses the GROC, as it is a universal outcome across all conditions, incorporating perceived changes in patients by considering all factors, which may explain the higher proportion of patients achieving the MCID reported for the GROC compared to the condition-specific measures in Table 2. Another limitation is that there were limited service- and patient-related variables available in this study, making it challenging to derive strong association models to explain the variations in the outcomes between facilities. We anticipate that the next service evaluation study will permit stronger inferences regarding facility variation, underpinned by a wider suite of metrics driven by the findings of this current study.

## 5. Conclusions

The findings demonstrated a substantial positive impact of the advanced physiotherapist-led services on overburdened public hospital specialist orthopaedic and neurosurgical outpatient services across Queensland, Australia. Potentially, this impact could be greater, given the observed disparities between facilities in discharge pathway outcomes, even following an adjustment for the differences in patient-related characteristics. While some significant service-related characteristics influencing these disparities between facilities were identified (duration of the management period, the initial triage category, patient nonattendance to review appointments, and medical specialist input during the management period) to be potentially addressed in future service planning, our findings highlighted the need for a more comprehensive collection of service- and patient-related metrics across facilities in the future. Subsequently, a new set of service evaluation metrics were described.

## Figures and Tables

**Table 1:** Secondary outcomes and potential explanatory variables (units/categories) included in the analysis. The condition specific, general health, and psychological measures were recorded at the initial consultation and discharge, with changes in these measures with respect to time representing the secondary patient-related clinical outcomes.

**Secondary Service—Related Outcomes and Explanatory Variables**
Outpatient Service (orthopaedic, neurosurgical)—indicates specialist medical outpatient service receiving the original patient referral.
Triage Category (1–3)—Patient referrals are categorised as urgent (category 1), semi-urgent (category 2) or nonurgent (category 3), with the recommended timeframes for an initial outpatient consultation within 30, 90, and 365 days, respectively.
Waiting Time (days)—time between specialist outpatient department receipt of initial referral and initial N/OPSC & MDS appointment.
Management Duration (days)—time between initial N/OPSC & MDS appointment and discharge.
Review Appointments (absolute number)—number of patients receiving an N/OPSC & MDS review appointment.
Non-attendance (yes/no)—number of patients not attending the final N/OPSC & MDS review appointment.
Multidisciplinary Referrals (number)—number of patients referred to multidisciplinary treatment services (may be one or more of the services, as clinically indicated).
Medical Specialist Case Discussion (yes/no)—number of patients for whom case discussion with a medical consultant was sought during the N/OPSC & MDS management period.
**Secondary Patient—Related Outcomes and Explanatory Variables**
Sociodemographic measures
Age (years)
Gender (male/female)
Socioeconomic Indexes for Areas (SEIFA) of Advantage/Disadvantage—score based on residential postcode with <1000 representing disadvantage) [11].
Condition-specific measures
Condition Managed (e.g., knee).
Pain Severity (score/100)—scored on a 100-mm visual analogue scale anchored by “0 No Pain” and “100 Severe Pain” [12] with a Minimal Clinically Important Difference (MCID) of 15 points [13,14].
Patient-Specific Functional Scale (PSFS) (score/10)—denoting the patient reported level of function [15] with a MCID of 2 points [16].
Oswestry Disability Index (ODI) (score/100)—self-reported disability for patients with thoracic/lumbar/Sacro-iliac joint (SIJ) conditions [17] and a MCID of 10 points [18].
Neck Disability Index (NDI) (score/100)—self-reported disability for patients with cervical conditions [19] and a MCID of 10 points [20].
Lower Extremity Functional Scale (LEFS) (score/80)—self-reported function for patients with hip/knee/foot/ankle conditions [21] and a MCID of 9 points [21].
Quick Disabilities of the Arm, Shoulder and Hand (QDASH) (score/100)—self-reported disability for patients with shoulder/elbow/wrist/hand conditions [22] and a MCID of 10 points [23].
General health measures
Body Mass Index (BMI) (km/m^2^).
Quality of Life Uni-scale (QOL) (score/100)—measured on a 100-mm visual analogue scale anchored by the terms “0 Lowest Quality” to “100 Highest Quality” [24].
Psychological measures
Pain Self-Efficacy Questionnaire (PSEQ) (score/60) [25] and a MCID of 10 points [16].
Depression, Anxiety and Stress Scale (DASS-21) with separate measures of depression (score/42), anxiety (score/42), and stress (score/42) [26].

**Table 2:** Service outcomes and explanatory variables presented as the number (*n*) and proportion (%) of patients within each category. The primary service outcome was a discharge pathway, dichotomised as either discharged from the service with no specialist medical review required (Discharged) or reinstated for specialist medical review (Specialist Review).

Variables	*n*	%
**Primary Service Outcome—Discharge Pathway**		
Discharged	20,004	69.4%
Cervical/Thoracic/Lumbar/Sacro-iliac	9784	77.8%
Shoulder/Elbow/Wrist/Hand	4528	66.9%
Hip/Knee/Ankle/Foot	5692	60.0%
Specialist Review	8819	30.6%
**Potential Explanatory Variables**		
Outpatient Service		
Orthopaedic	23,386	79.8%
Neurosurgical	5933	20.2%
Triage Category		
1	237	0.8%
2	10,451	35.6%
3	18,631	63.5%
Waiting Time		
<2 weeks	720	2.5%
<1 month	3133	10.7%
1 to 2 months	6439	22.0%
2 to 3 months	4171	14.2%
>3 months	14,492	49.4%
Management Duration		
1 Day	5163	17.6%
<2 weeks	552	1.9%
<1 month	500	1.7%
1 to 2 months	1145	3.9%
2 to 3 months	2432	8.3%
>3 months	19,161	65.4%
Review Appointments	17,826	60.8%
Nonattendance	4666	15.9%
Multidisciplinary Referrals	19,788	73.5%
Medical Specialist Case Discussion during N/OPSC Management	4462	15.2%

**Table 3:** Clinical outcomes and potential patient-related explanatory variables recorded at the initial service assessment and at discharge presented as means (95% confidence interval (CI)) or proportions (%), mean change between initial assessment and discharge, and the proportion of cases achieving a Minimal Clinically Important Difference (MCID).

Variables	Initial Assessment			Discharge Assessment							
	*n*	Mean or %	95% CI	*n*	Mean	95% CI	Mean Change	MCID	*T*-Test	Sig	% Meeting MCID
**Primary Clinical Outcome—Global Rating of Change (GROC)**											
Cervical/Thoracic/Lumbar/Sacro-iliac				2999	2.1	(2.1–2.2)					68.6% *
Hip/Knee/Ankle/Foot				4274	1.9	(1.9–2.0)					65.7% *
Shoulder/Elbow/Wrist/Hand				2859	2.5	(2.4–2.6)					74.1% *
Combined Regions				10,132	2.2	(2.1–2.2)					68.9% *
**Secondary Clinical Outcomes and Potential Patient-Related Explanatory Variables**											
Condition Specific Measures of Pain, Function, and Disability											
Pain Severity (score/100)	23,486	58	(58–58)	10,050	39	(38–39)	−18.3	15	63.64	<0.001	50.2%
PSFS (score/10)	9696	4	(4–4)	4248	6	(6–6)	2.3	2	57.41	<0.001	59.9%
ODI (score/100)	7159	42	(42–43)	2493	32	(31–33)	−9.6	10	−27.45	<0.001	42.6%
NDI (score/100)	2088	41	(40–41)	743	30	(28–31)	−9.3	10	−15.47	<0.001	43.1%
LEFS (score/80)	8210	35	(34–35)	3971	45	(44–45)	9.8	9	36.97	<0.001	48.9%
QDASH (score/100)	5604	51	(50–51)	2663	32	(31–33)	−17.4	10	−39.92	<0.001	61.3%
General Health and Psychological Measures											
BMI (km/m^2^)	18,000	30.5	(30.3–30.6)	5991	30.8	(30.6–31)	0.3	N/A	1.59	0.112	N/A
QOL (score/100)	23,385	60	(59–60)	9904	69	(68–69)	6.5	N/A	23.41	<0.001	32.9%
PSEQ (score/60)	23,436	31	(31–32)	9301	40	(40–40)	7.0	10	46.57	<0.001	37.6%
DASS—Depression (score/42)	22,298	12	(12–12)	8814	9	(9–9)	−2.1	N/A	−21.47	<0.001	N/A
DASS—Anxiety (score/42)	22,311	9	(9–9)	8817	7	(7–7)	−1.0	N/A	−12.07	<0.001	N/A
DASS—Stress (score/42)	22,319	14	(14–14)	8818	11	(10–11)	−2.0	N/A	−20.28	<0.001	N/A
Sociodemographic Measures				Condition Managed % (*n*)							
Age (years)	29,084	53.9	(53.7–54.1)	Neck 3007 (10.3%), Thoracic 193 (0.7%), Lumbar/Sacroiliac Joint 9681 (33%), Shoulder 5854 (20%), Elbow 333 (1.1%), Wrist/Hand 677 (2.3%), Hip 1291 (4.4%), Knee 7061 (24.1%), Ankle/Foot 1222 (4.2%)							
SEIFA (points)	28,384	988.21	(987.5–988.9)								
Gender—Male	13,703	47.0%									
Gender—Female	15,467	53.0%	-								

* Global Rating of Change MCID considered as score of 2 or greater. Patient Specific Functional Scale (PSFS); Oswestry Disability Index (ODI); Neck Disability Index (NDI); Lower Extremity Functional Scale (LEFS); Quick Disabilities of Arm, Shoulder and Hand (QDASH); Body Mass Index (BMI); Quality of Life (QOL); Patient Self-Efficacy Questionnaire (PSEQ); Depression, Anxiety and Stress Scale (DASS); and Socioeconomic Indexes for Areas (SEIFA).

**Table 4:** Three hierarchical binomial regression models for the primary service outcome (Discharged) evaluating the relationship between the discharge pathway and Neurosurgical and Orthopaedic Physiotherapy Screening Clinic and Multi-disciplinary Service (N/OPSC & MDS) facility only (Model 1), adjusted for the patient-related variables (Model 2), and adjusted for the service-related variables (Model 3). Significant service variables in the final model included management duration, triage category, nonattendance, and medical specialist input. Shaded cells represent sites with significant variance from the referent facility (Facility 1). OR: adds ratio.

Variable	Category	Model 1: Facility Only		Model 2: Facility and Patient Variables		Model 3: Facility, Patient, and Service Variables	
	
		OR (95% CI)	Sig.	OR (95% CI)	Sig.	OR (95% CI)	Sig.
**Service facilities**	1	Referent					
	2	1.82 (1.53–2.18)	<0.01	2.01 (1.63–2.47)	<0.01	2.04 (1.6–2.6)	<0.01
	3	0.92 (0.49–1.71)	0.78	1.21 (0.59–2.48)	0.6	2.92 (1.29–6.6)	0.01
	4	0.97 (0.8–1.17)	0.72	1.51 (0.78–2.94)	0.22	3.43 (1.55–7.59)	<0.01
	5	1.98 (1.55–2.52)	<0.01	1.97 (1.44–2.69)	<0.01	2.77 (1.95–3.93)	<0.01
	6	1.07 (0.93–1.24)	0.32	1.04 (0.81–1.32)	0.78	1.07 (0.81–1.4)	0.64
	7	0.28 (0.24–0.33)	<0.01	0.35 (0.29–0.42)	<0.01	0.28 (0.23–0.34)	<0.01
	8	1.14 (0.96–1.37)	0.15	1.44 (1.15–1.8)	<0.01	1.28 (1–1.64)	0.05
	9	1.17 (1.01–1.36)	0.04	0.88 (0.71–1.1)	0.27	0.72 (0.56–0.92)	0.01
	10	0.45 (0.38–0.52)	<0.01	0.27 (0.16–0.45)	<0.01	0.27 (0.15–0.47)	<0.01
	11	1.28 (1.09–1.51)	<0.01	0.94 (0.74–1.18)	0.58	1.14 (0.86–1.52)	0.35
	12	0.37 (0.31–0.44)	<0.01	0.44 (0.35–0.54)	<0.01	0.39 (0.3–0.49)	<0.01
	13	2.36 (1.98–2.81)	<0.01	2.17 (1.62–2.9)	<0.01	1.97 (1.44–2.69)	<0.01
	14	1.54 (1.31–1.82)	<0.01	1.03 (0.82–1.28)	0.83	1.89 (1.44–2.48)	<0.01
	15	1.51 (1.17–1.96)	<0.01	0.81 (0.58–1.14)	0.23	2.25 (1.5–3.37)	<0.01
	16	3.99 (1.81–8.78)	<0.01				
	17	1.67 (1.41–1.97)	<0.01	1.91 (1.57–2.33)	<0.01	1.71 (1.36–2.17)	<0.01
	18	0.77 (0.66–0.89)	<0.01	0.89 (0.73–1.07)	0.22	1 (0.81–1.24)	0.99
	19	1.05 (0.89–1.22)	0.58	1.41 (1.16–1.7)	<0.01	1.52 (1.22–1.91)	<0.01
	20	0.53 (0.43–0.65)	<0.01	0.51 (0.38–0.67)	<0.01	0.45 (0.33–0.62)	<0.01
**Patient-Related Variables**							
Age (years)	0–19			Referent		Referent	
	20–39			0.78 (0.59–1.04)	0.09	0.67 (0.5–0.92)	0.01 *
	40–59			0.82 (0.62–1.07)	0.15	0.71 (0.53–0.96)	0.02 *
	60–79			0.69 (0.52–0.9)	0.01 *	0.63 (0.47–0.85)	<0.01 *
	80+			0.81 (0.57–1.14)	0.23	0.85 (0.58–1.24)	0.39
Sex	Female			1.22 (1.13–1.31)	<0.01 *	1.11 (1.02–1.2)	0.02 *
Body Mass Index				1 (1–1.01)	0.51	1 (0.99–1)	0.43
Pain Severity				0.98 (0.98–0.98)	<0.01 *	0.98 (0.98–0.98)	<0.01 *
Quality of Life				1.01 (1.01–1.01)	<0.01 *	1.01 (1–1.01)	<0.01 *
SEIFA Score				1 (1–1)	0.13	1 (1–1)	0.35
Condition Managed	Spinal				<0.01 *		<0.01 *
	Upper Limb			0.55 (0.49–0.62)	<0.01 *	0.52 (0.46–0.59)	<0.01 *
	Lower Limb			0.4 (0.35–0.44)	<0.01 *	0.38 (0.34–0.43)	<0.01 *
**Service-Related Variables**							
Waiting Time	<2 w						0.2
	<1 m					0.83 (0.64–1.09)	0.18
	1 to 2 m					0.81 (0.63–1.05)	0.11
	2 to 3 m					0.89 (0.68–1.17)	0.4
	>3 m					0.77 (0.6–1)	0.05 *
Management Duration	1 Day						<0.01 *
	<2 w					0.62 (0.44–0.88)	0.01 *
	<1 m					1.17 (0.83–1.65)	0.36
	1 to 2 m					1.92 (1.51–2.46)	<0.01 *
	2 to 3 m					2.48 (2.05–2.99)	<0.01 *
	>3 m					2.69 (2.34–3.08)	<0.01 *
Triage Category	Category 3						<0.01
	Category 1					0.48 (0.3–0.77)	<0.01 *
	Category 2					0.87 (0.78–0.97)	0.01 *
Nonattendance to final review	Not Attended					1.81 (1.59–2.06)	<0.01 *
Medical Specialist Case discussion Input	Yes					0.16 (0.15–0.18)	<0.01 *

* *p* < 0.05.

**Table 5:** Three hierarchical binomial regression models for the primary clinical outcome (Responder) evaluating the relationship between the clinical outcome and N/OPSC & MDS facility only (Model 1), adjusted for the patient-related variables (Model 2), and adjusted for service-related variables (Model 3). Significant service variables in the final model included management duration, triage category, nonattendance, and specialist input. Shaded cells represent sites with significant variance from the referent facility (Facility 10).

Variable	Category	Model 1: Facility Only		Model 2: Facility and Patient Variables		Model 3: Facility, Patient, and Service Variables	
	
		OR (95% CI)	Sig.	OR (95% CI)	Sig.	OR (95% CI)	Sig.
**Service facilities**	10	Referent					
	1	1.88 (1.45–2.45)	<0.01	2.52 (1.32–4.81)	0.01	1.88 (0.93–3.78)	0.08
	2	1.62 (1.25–2.09)	<0.01	2.13 (1.12–4.03)	0.02	1.41 (0.71–2.81)	0.33
	3	0.78 (0.32–1.88)	0.57	1.23 (0.4–3.81)	0.72	1.11 (0.33–3.72)	0.87
	4	0.91 (0.68–1.22)	0.51	0.95 (0.32–2.86)	0.93	0.97 (0.3–3.11)	0.96
	5	1.77 (1.25–2.5)	<0.01	2.96 (1.47–5.97)	<0.01	2.18 (1.03–4.59)	0.04
	6	0.48 (0.37–0.63)	<0.01	0.77 (0.38–1.57)	0.48	0.48 (0.23–1.02)	0.06
	7	0.59 (0.47–0.75)	<0.01	0.74 (0.4–1.4)	0.36	0.44 (0.22–0.87)	0.02
	8	1.51 (1.16–1.95)	<0.01	2.16 (1.13–4.12)	0.02	1.33 (0.67–2.66)	0.42
	9	1.47 (1.12–1.92)	0.01	1.53 (0.77–3.03)	0.22	1.03 (0.5–2.14)	0.94
	11	1.26 (0.9–1.77)	0.18	1.79 (0.86–3.71)	0.12	1.31 (0.6–2.84)	0.5
	12	0.73 (0.57–0.94)	0.01	0.87 (0.46–1.66)	0.67	0.64 (0.32–1.27)	0.2
	13	0.71 (0.56–0.91)	0.01	1.03 (0.53–2)	0.94	0.63 (0.31–1.29)	0.21
	14	0.72 (0.52–0.99)	0.04	0.88 (0.44–1.76)	0.71	0.92 (0.43–1.94)	0.82
	15	3.54 (0.44–28.56)	0.24	1.92 (0.18–20.22)	0.59	1.11 (0.1–11.84)	0.93
	16	1 (0.3–3.28)	1				
	17	1.47 (1.16–1.85)	<0.01	1.7 (0.91–3.2)	0.1	0.85 (0.43–1.69)	0.64
	18	0.71 (0.57–0.89)	<0.01	0.94 (0.5–1.77)	0.85	0.67 (0.34–1.31)	0.24
	19	1.08 (0.85–1.36)	0.54	1.64 (0.88–3.06)	0.12	1.33 (0.68–2.59)	0.41
	20	0.57 (0.43–0.75)	<0.01	0.87 (0.44–1.72)	0.69	0.45 (0.22–0.94)	0.03
**Patient-Related Variables**							
Age (years)	0–19			Referent		Referent	
	20–39			0.34 (0.2–0.59)	<0.01 *	0.29 (0.16–0.52)	<0.01 *
	40–59			0.31 (0.18–0.53)	<0.01 *	0.25 (0.14–0.44)	<0.01 *
	60–79			0.32 (0.18–0.55)	<0.01 *	0.26 (0.15–0.46)	<0.01 *
	80+			0.28 (0.15–0.51)	<0.01 *	0.23 (0.12–0.43)	<0.01 *
Sex	Female			1.33 (1.19–1.49)	<0.01 *	1.31 (1.16–1.47)	<0.01 *
Body Mass Index				1 (0.99–1)	0.33	1 (0.99–1)	0.29
Pain Severity				0.98 (0.98–0.99)	<0.01 *	0.98 (0.98–0.99)	<0.01 *
Quality of Life				1.01 (1–1.01)	<0.01 *	1.01 (1–1.01)	<0.01 *
SEIFA Score				1 (1–1)	<0.01 *	1 (1–1)	0.03 *
Condition Managed	Spinal				<0.01 *		<0.01 *
	Upper Limb			1.31 (1.1–1.55)	<0.01 *	1.35 (1.13–1.61)	<0.01 *
	Lower Limb			0.75 (0.65–0.88)	<0.01 *	0.79 (0.68–0.93)	0.01 *
**Service-Related Variables**							
Waiting Time	<2 w						0.00 *
	<1 m					0.85 (0.6–1.22)	0.39
	1 to 2 m					0.79 (0.55–1.12)	0.18
	2 to 3 m					0.69 (0.48–1)	0.05 *
	>3 m					0.57 (0.4–0.8)	<0.01 *
Management Duration	1 Day						<0.01 *
	<2 w					1.08 (0.27–4.39)	0.91
	<1 m					6.7 (2.27–19.81)	<0.01 *
	1 to 2 m					5.7 (2.8–11.59)	<0.01 *
	2 to 3 m					7.35 (3.87–13.95)	<0.01 *
	>3 m					6.64 (3.59–12.29)	<0.01 *
Triage Category	Category 3						0.03 *
	Category 1					1.04 (0.46–2.37)	0.92
	Category 2					1.25 (1.06–1.48)	0.01 *
Nonattendance to final review	Not Attended					0.62 (0.38–1.02)	0.06
Medical Specialist Case discussion Input	Yes					0.33 (0.28–0.38)	<0.01 *

* *p* < 0.05.

## Data Availability

The dataset from this study is not publicly available due to the data having been collated from multiple hospital health services, each with individual data custodians that require further approval for access. Please contact the first author (Maree.Raymer@health.qld.gov.au) regarding any data requests.

## Appendix A. Revised State-Wide Dataset

The changes to the standardised dataset, from the results of this study, literature review, and consultation within the N/OPSC & MDS, are shown in Appendix A Table A1. New and modified measures, and those where additional response options were added, are identified. Appendix A Table A2 provides the definitions of the performance indicators against which the data is reported.

**Table A1:** Revised standardised data set. New and modified measures, and those where additional response options were added, are identified.

**Referral Details:**	
Patient details: • Unit Record number • Date of birth • Residential postcode • Surname • First initial • Sex	Referral details: • Date referral received (in SOPD) • Date PSC initial appointment attended • Specific long-wait initiative (New) • Interpreter required (New) • Triage category • Referral source (Additional response options) • Primary region (Additional response options)
**Demographic Information:**	
• Country of birth (New) • ATSI status (New) • Employment status (New) • Level of education (New)	• Smoking status (New) • Comorbidities (New) • Physical activity (New) • Analgesic consumption (New)
**Clinical Outcomes (completed at intake and discharge from service):**	
• NRS Pain (Modified Measure) • STarT MSK tool (initial only) (New) • PSEQ-2 (Modified Measure) • AQoL-4D (Modified Measure) • Height/Weight • Region-specific questionnaire (one per patients according to primary region of referral) ⚬ Oswestry Disability Index, Neck Disability Index, QuickDASH, Lower Extremity Functional Scale, LDF-TMD-Jaw Function Scale (New) • GROC (at discharge only)	
**Episode of Care Details:**	
• Investigations initiated (New) ⚬ Type of investigations initiated • Interventions initiated (New) ⚬ Type of interventions initiated • Other AH referrals initiated ⚬ Type of AH referrals initiated ⚬ AH referral funding sources (New)	
**PSC Discharge Outcome:**	
• Discharge date • Number of PSC review appointments • Discharge outcome: (Modified Measure) ⚬ A. No further SOPD follow-up required ⚬ B. Further SOPD follow-up required ▪ Discharge follow-up reason ▪ Why patient returned to waitlist ⚬ C. Due to nonattendance ▪ Patient remains on SOPD waitlist? ⚬ D. Administrative (not seen by PSC) ▪ Patient remains on SOPD waitlist? • Medical Consultant case discussion during PSC management ⚬ Reason/s for medical consultant case discussion (New) • Telehealth Use (New) • Adverse event reported (New) ⚬ Riskman incident number	

SOPD: Specialist Outpatient Department; PSC: Physiotherapy Screening Clinic; ATSI: Aboriginal and/or Torres Strait Islander; NRS: Numerical Rating Scale; PSEQ-2: Pain Self-Efficacy Questionnaire-2 items; AQoL-4D: Assessment of Quality of Life-4 Dimensions; QuickDASH: Quick Disabilities of the Arm, Shoulder and Hand; LDF-TMD-Jaw Function Scale: Limitations of Daily Function Questionnaire for Patients with Tempero-Mandibular Disorder; AH: Allied Health; and GROC: Global Rating of Change.

**Table A2:** Performance indicators against which the standardised dataset is reported.

Indicator	Definition	Numerator	Denominator
**1. Service Profile:**			
1.1 PSC Referral Source	Proportion of patients from each referral source.	Number of patients referred by each nominated referral source.	Total eligible patients
1.2 Primary Region	Proportion of patients referred for conditions of each body region.	Number of patients referred by each nominated primary region.	Total eligible patients
1.3 Referral Categorisation	Proportion of patients referred in each triage urgency Category.	Number of patients referred by each triage urgency category.	Total eligible patients
1.4 STarT MSK Risk Stratification Tool	Proportion of patients in each STarT MSK risk category (low, medium, high).	Number of patients in each risk category (low, medium, high).	Total eligible patients
**2. Activity/Throughput:**			
2.1 Number of patients discharged from PSC	Number of submitted records.	N/A	N/A
2.2 Average waiting time to initial PSC appointment	Average waiting time (days) from date referral received by SOPD to initial PSC appointment. (First Appointment Date—Referral Date).	N/A	N/A
2.3 Proportion of patients seen within Category-specific timeframes	Proportion of PSC patients that attended their initial appointment within the triage category-specific target timeframe. (Urgent—<30 days, Semi-Urgent—<90 days, and Routine—<365 days).	Number of patients where wait difference (days) is less than predetermined time of triage category they were assigned against.	Number of eligible forms submitted for each triage category
2.4 Average length of stay	Average length of patient admission to the PSC (PSC Discharge Date—date of PSC initial appointment).	N/A	N/A
2.5 Ratio of new: review patient visits	Ratio of the number of new visits to the PSC compared to the number of completed review visits.	Number of new patient visits.	Number of review patient visits
**3. Episode of Care:**			
3.1 Nonsurgical management	Proportion of PSC patients who are referred to any nonsurgical management as part of their admission with the service.	Number patients where other AH professions referrals have been initiated (YES).	Number eligible forms submitted
3.2 Referral Types (nonsurgical management)	Proportion of PSC patients who consent to referral to each Allied Health profession for nonsurgical management as part of their admission with the service.	Number of patients referred to each referral type (Physiotherapy, Nutrition and Dietetics, Occupational Therapy, Psychology, Pharmacy, and Other).	Number eligible forms submitted
3.3 AH Referrals and funding sources	Proportion of PSC patients referred to Allied Health services based on funding source.	Number of patients referred to each funding source type for each individual AH referral.	Number patients referred to each AH referral type
3.4 Further investigations initiated	Proportion of PSC patients who had investigations initiated as part of their admission with the service.	Number of patients who had investigations initiated by PSC.	Number of eligible forms submitted
3.5 Further interventions initiated	Proportion of PSC patients who had interventions initiated as part of their admission with the service.	Number of patients who had interventions initiated by PSC.	Number of eligible forms submitted
3.6 Medical Consultant Case Discussion	Proportion of patient cases in which the Service Leader sought case discussion with a Medical Consultant during the patient’s admission with the PSC.	Number of patient where medical consultant case discussion was sought.	Number of eligible forms submitted
3.8 Telehealth Use	Proportion of PSC patients that have received any type of clinical services delivered via telehealth (videoconferencing) as part of their admission with the service.	Number patients who received clinical services via telehealth.	Number of eligible forms submitted
3.9 Adverse Events	Proportion of PSC patients who experienced an adverse clinical event in which harm occurred (actual) or could have occurred (potential or near miss) as part of the clinical care in the service	Number of patients with adverse events.	Number of eligible forms submitted
**4. Patient-reported Outcome Measures (PROMs):**			
4.1 Global Outcome Scores—MCID	Proportion of PSC patients who achieve a minimal clinically important difference (MCID) in their condition and includes Pain NRS, PSEQ-2, and GROC	Number of patients who achieved MCID for that specific outcome score (requires pre- and post-outcomes to be entered, except for GROC—discharge only).	Number of eligible forms submitted
4.2 Region-specific outcome scores—MCID	Proportion of PSC patients who achieve a minimal clinically important difference (MCID) in their condition on measures which specifically take into consideration the body region for which they have sought treatment.	Number of patients who have achieved MCID for that specific outcome score (requires pre- and post-outcomes to be entered).	Number of eligible forms submitted where corresponding body region is checked
4.3 Global Outcome Scores—Initial and Discharge	This indicator provides the scores obtained at either initial assessment and/or discharge with respect to measures of pain, self-efficacy, and overall improvement.	Number of patients with valid score for either initial and/or discharge for global outcomes.	Number of eligible forms submitted
Region-specific outcome scores—Initial and Discharge	This indicator examines the region-specific scores obtained at either initial assessment and/or discharge with respect to the body region for which they have sought treatment.	# of patients with valid score for either initial and/or discharge for their respective region-specific questionnaire.	Number eligible forms submitted where corresponding body region is checked
**5. Discharge Outcomes:**			
5.1 Discharge from PSC following initial assessment	Proportion of patients that are discharged from the PSC following their initial assessment. Excludes patients discharged due to administrative reasons.	Number of patients where LOS (Discharge Date—PSC Initial Appointment) is ≤2 days.	Number of eligible forms submitted
5.2 Patients discharged with no further SOPD follow-up (Discharge Outcome A)	Proportion of PSC patients who are removed from the SOPD waitlist on discharge from the service.Excludes patients removed from the SOPD waitlist following Discharge due to Nonattendance or Administrative Discharges.	Number patients discharged with no further medical follow-up (Discharge Outcome A).	Number of eligible forms submitted
5.3 Patients discharged with further SOPD follow-up (Discharge Outcome B)	Proportion of PSC patients who will require to be returned/remain on the SOPD waitlist on discharge from the service. Excludes patients remaining on the SOPD waitlist following Discharge due to Nonattendance or Administrative Discharges.	Number of patients discharged with further medical follow-up required (Discharge Outcome B).	Number of eligible forms submitted
5.4 Patients discharged due to nonattendance (Discharge Outcome C)	Proportion of PSC patients discharged as a direct result of nonattendance of a scheduled review appointment.	Number patients who are discharged due to nonattendance (Discharge Outcome C).	Number eligible forms submitted
5.5 PSC Administrative Discharge (Discharge Outcome D)	Proportion of PSC patients referred to the PSC but were discharged from the service without attending an initial appointment (e.g., failure to attend, declined appointment, etc.).	Number of patients discharged as an administration discharge (Discharge Outcome D).	Number eligible forms submitted
5.6 Total patients removed from SOPD waitlists	Proportion of all patients who were removed from the SOPD waitlist upon discharge from the N/OPSC or OSiP service.	Summation of number of patients with proposed discharge status options checked. ((A) No further SOPD follow-up + (Discharge C and D, where patient is removed from SOPD waitlist).	Number eligible forms submitted

N/A: Not Applicable, PSC Physiotherapy Screening Clinic, AH: Allied Health, and LOS: Length of Stay, OSiP: Orthopaedic Screening in Primary Care.

## References

[1] Moretto N., Comans T.A., Chang A.T., O’Leary S.P., Osborne S., Carter H.E., Smith D., Cavanagh T., Blond D., Raymer M. (2019). Implementation of simulation modelling to improve service planning in specialist orthopaedic and neurosurgical outpatient services. Implement. Sci..

[2] Cottrell M.A., O’Leary S.P., Swete-Kelly P., Elwell B., Hess S., Litchfield M.A., McLoughlin I., Tweedy R., Raymer M., Hill A.J. (2018). Agreement between telehealth and in-person assessment of patients with chronic musculoskeletal conditions presenting to an advanced-practice physiotherapy screening clinic. Musculoskelet. Sci. Pract..

[3] Scopaz K.A., Piva S.R., Wisniewski S., Fitzgerald G.K. (2009). Relationships of fear, anxiety, and depression with physical function in patients with knee osteoarthritis. Arch. Phys. Med. Rehabil..

[4] Hurley M., Dickson K., Hallett R., Grant R., Hauari H., Walsh N., Stansfield C., Oliver S. (2018). Exercise interventions and patient beliefs for people with hip, knee or hip and knee osteoarthritis: A mixed methods review. Cochrane Database Syst. Rev..

[5] Rymaszewski L.A., Sharma S., McGill P.E., Murdoch A., Freeman S., Loh T. (2005). A team approach to musculo-skeletal disorders. Ann. R. Coll. Surg. Engl..

[6] Chang A.T., Gavaghan B., O’Leary S., McBride L.J., Raymer M. (2018). Do patients discharged from advanced practice physiotherapy-led clinics re-present to specialist medical services?. Aust. Health Rev..

[7] Comans T., Raymer M., O’Leary S., Smith D., Scuffham P. (2014). Cost-effectiveness of a physiotherapist-led service for orthopaedic outpatients. J. Health Serv. Res. Policy.

[8] Partington A., Chew D.P., Ben-Tovim D., Horsfall M., Hakendorf P., Karnon J. (2017). Screening for important unwarranted variation in clinical practice: A triple-test of processes of care, costs and patient outcomes. Aust. Health Rev..

[9] Australian Commission on Safety and Quality in Health Care (ACSQHC) (2013). Medical Practice Variation: Background Paper.

[10] Kamper S.J., Maher C.G., Mackay G. (2009). Global rating of change scales: A review of strengths and weaknesses and considerations for design. J. Man. Manip. Ther..

[11] What is SEIFA? Canberra: Australian Bureau of Statistics. http://www.abs.gov.au/ausstats/abs@.nsf/Lookup/2033.0.55.001main+features42011.

[12] Fries J., Spitzer P., Kranes G., Holman H. (1980). Measurement of Patient Outcome in Arthritis. Arthritis Rheum..

[13] Tashjian R.Z., Deloach J., Porucznik C.A., Powell A.P. (2009). Minimal clinically important differences (MCID) and patient acceptable symptomatic state (PASS) for visual analog scales (VAS) measuring pain in patients treated for rotator cuff disease. J. Shoulder Elbow Surg..

[14] Tubach F., Ravaud P., Baron G., Falissard B., Logeart I., Bellamy N., Bombardier C., Felson D., Hochberg M., van der Heijde D. (2005). Evaluation of clinically relevant changes in patient reported outcomes in knee and hip osteoarthritis: The minimal clinically important improvement. Ann. Rheum. Dis..

[15] Stratford P., Gill C., Westaway M., Binkley J. (1995). Assessing disability and change on individual patients: A report of a patient specific measure. Physiother. Can..

[16] Maughan E.F., Lewis J.S. (2010). Outcome measures in chronic low back pain. Eur. Spine J..

[17] Lauridsen H.H., Hartvigsen J., Manniche C., Korsholm L., Grunnet-Nilsson N. (2006). Responsiveness and minimal clinically important difference for pain and disability instruments in low back pain patients. BMC Musculoskelet. Disord..

[18] Hagg O., Fritzell P., Nordwall A., Swedish Lumbar Spine Study G. (2003). The clinical importance of changes in outcome scores after treatment for chronic low back pain. Eur. Spine J..

[19] Vernon H., Mior S. (1991). The neck disability index: A study of reliability and validity. J. Manip. Physiol. Ther..

[20] Riddle D.L., Stratford P.W. (1998). Use of generic versus regional specific functional status measures on patients with cervical spine disorders. Phys. Ther..

[21] Binkley J.M., Stratford P.W., Lott S.A., Riddle D.L. (1999). The Lower Extremity Functional Scale (LEFS): Scale development, measurement properties, and clinical application. North American Orthopaedic Rehabilitation Research Network. Phys. Ther..

[22] Beaton D.E., Wright J.G., Katz J.N. (2005). Development of the QuickDASH: Comparison of three item-reduction approaches. J. Bone Jt. Surg. Am..

[23] Fan Z.J., Smith C.K., Silverstein B.A. (2008). Assessing validity of the QuickDASH and SF-12 as surveillance tools among workers with neck or upper extremity musculoskeletal disorders. J. Hand Ther..

[24] Spitzer W.O., Dobson A.J., Hall J., Chesterman E., Levi J., Shepherd R., Battista R.N., Catchlove B.R. (1981). Measuring the quality of life of cancer patients: A concise QL-index for use by physicians. J. Chronic Dis..

[25] Nicholas M. (2007). The Pain Self-Efficacy Questionnaire: Taking pain into account. Eur. J. Pain..

[26] Henry J.D., Crawford J.R. (2005). The short-form version of the Depression Anxiety Stress Scales (DASS-21): Construct validity and normative data in a large non-clinical sample. Br. J. Clin. Psychol..

[27] Liu Q., Li C., Wanga V., Shepherd B.E. (2018). Covariate-adjusted Spearman’s rank correlation with probability-scale residuals. Biometrics.

[28] Akoglu H. (2018). User’s guide to correlation coefficients. Turk. J. Emerg. Med..

[29] Dancey C.P., Reidy J. (2007). Statistics without Maths for Psychology.

[30] Field A.P. (2009). Discovering Statistics Using SPSS: (and Sex and Drugs and Rock ‘n’ Roll).

[31] Box G.E.P., Tidwell P.W. (1962). Transformation of the independent variables. Technometrics.

[32] Gray J.B., Woodall W.H. (1994). The Maximum Size of Standardized and Internally Studentized Residuals in Regression Analysis. Am. Stat..

[33] Standfield L., Comans T., Raymer M., O’Leary S., Moretto N., Scuffham P. (2016). The Efficiency of Increasing the Capacity of Physiotherapy Screening Clinics or Traditional Medical Services to Address Unmet Demand in Orthopaedic Outpatients: A Practical Application of Discrete Event Simulation with Dynamic Queuing. Appl. Health Econ. Health Policy.

[34] Comans T.A., Chang A.T., Standfield L., Knowles D., O’Leary S., Raymer M. (2017). The development and practical application of a simulation model to inform musculoskeletal service delivery in an Australian public health service. Oper. Res. Health Care.

[35] Mulley A.J. (2010). Improving productivity in the NHS. BMJ.

[36] Fehring T.K. (2016). AAHKS Risk Adjustment Initiative: Why Is It Important?. J. Arthroplast..

[37] Yen S.C., Corkery M.B., Chui K.K., Manjourides J., Wang Y.C., Resnik L.J. (2015). Risk Adjustment for Lumbar Dysfunction: Comparison of Linear Mixed Models With and Without Inclusion of Between-Clinic Variation as a Random Effect. Phys. Ther..

[38] Sangha O., Stucki G., Liang M.H., Fossel A.H., Katz J.N. (2003). The Self-Administered Comorbidity Questionnaire: A new method to assess comorbidity for clinical and health services research. Arthritis Rheum..

[39] Fennelly O., Blake C., Desmeules F., Stokes D., Cunningham C. (2018). Patient-reported outcome measures in advanced musculoskeletal physiotherapy practice: A systematic review. Musculoskelet. Care.

[40] Nicholas M.K., McGuire B.E., Asghari A. (2015). A 2-item short form of the Pain Self-efficacy Questionnaire: Development and psychometric evaluation of PSEQ-2. J. Pain.

[41] Hill J.C., Garvin S., Chen Y., Cooper V., Wathall S., Saunders B., Lewis M., Protheroe J., Chudyk A., Dunn K.M. (2020). Stratified primary care versus non-stratified care for musculoskeletal pain: Findings from the STarT MSK feasibility and pilot cluster randomized controlled trial. BMC Fam. Pract..

[42] Clement R.C., Welander A., Stowell C., Cha T.D., Chen J.L., Davies M., Fairbank J.C., Foley K.T., Gehrchen M., Hagg O. (2015). A proposed set of metrics for standardized outcome reporting in the management of low back pain. Acta Orthop..

[43] Williams K., Sansoni J., Morris D., Grootemaat P., Thompson C. (2016). Patient-Reported Outcome Measures: Literature Review.

[44] ICHOM Hip & Knee Osteoarthritis Data Collection Reference Guide. https://ichom.org/files/medical-conditions/hip-knee-osteoarthritis/hip-knee-osteoarthritis-reference-guide.pdf.

